# Relative judgment theory and the mediation of facial recognition: Implications for theories of eyewitness identification

**DOI:** 10.1186/s41235-016-0014-7

**Published:** 2016-11-05

**Authors:** Ryan M. McAdoo, Scott D. Gronlund

**Affiliations:** Department of Psychology, University of Oklahoma, Norman, OK 73071 USA

**Keywords:** Eyewitness identification, Facial recognition, One-high-threshold (1HT) model, Signal-detection theory (SDT), Relative and absolute judgments

## Abstract

Many in the eyewitness identification community believe that sequential lineups are superior to simultaneous lineups because simultaneous lineups encourage inappropriate choosing due to promoting comparisons among choices (a relative judgment strategy), but sequential lineups reduce this propensity by inducing comparisons of lineup members directly to memory rather than to each other (an absolute judgment strategy). Different versions of the relative judgment theory have implicated both discrete-state and continuous mediation of eyewitness decisions. The theory has never been formally specified, but (Yonelinas, J Exp Psychol Learn Mem Cogn 20:1341–1354, 1994) dual-process models provide one possible specification, thereby allowing us to evaluate how eyewitness decisions are mediated. We utilized a ranking task (Kellen and Klauer, J Exp Psychol Learn Mem Cogn 40:1795–1804, 2014) and found evidence for continuous mediation when facial stimuli match from study to test (Experiment 1) and when they mismatch (Experiment 2). This evidence, which is contrary to a version of relative judgment theory that has gained a lot of traction in the legal community, compels reassessment of the role that guessing plays in eyewitness identification. Future research should continue to test formal explanations in order to advance theory, expedite the development of new procedures that can enhance the reliability of eyewitness evidence, and to facilitate the exploration of task factors and emergent strategies that might influence when recognition is continuously or discretely mediated.

## Significance statement

It is vital to understand the processes that underlie eyewitness identification because faulty eyewitness identification is a primary contributor to wrongful convictions (contributing to 72 % of the wrongful convictions litigated by the Innocence Project). Our experiments combine elements from basic memory theory with the important applied question of how memory for a perpetrator is evaluated by an eyewitness. Using a paradigm that has a clear resemblance to a police photo lineup, our studies enhance our understanding of the theory underlying eyewitness identification by providing evidence that facial recognition is continuously mediated. Not only do these results contradict theories of eyewitness identification that include a non-diagnostic guessing process, the results point to existing theories that are better suited to handle the data.

## Background

Faulty eyewitness identification (ID) has contributed to the wrongful conviction of hundreds of innocent men and women, playing a role in 72 % of DNA exoneration cases litigated by the Innocence Project (Innocence Project 2015). Understanding the factors that influence these mistaken IDs is of great theoretical and practical interest. One such factor concerns the use of simultaneous or sequential lineups, but our goal here is not to evaluate the empirical evidence marshaled for and against sequential lineups (see Clark, Moreland, & Gronlund 2014; Steblay, Dysart, & Wells 2011; for a review, see Gronlund, Mickes, Wixted, & Clark 2015). Rather, our goal is to formalize and test theoretical conceptualizations that have been proposed to explain the functioning of simultaneous and sequential lineups.

The typical procedure in studies of eyewitness ID involves presenting participants with a mock crime scenario (usually a video) followed by a delay. Participants are then shown either a target present lineup, containing the guilty suspect from the video and five known innocents (fillers), or a target absent lineup, containing a designated innocent suspect and five fillers. If the guilty suspect is identified from a target present lineup, it is counted as a correct ID. If the innocent suspect is identified from a target absent lineup, it is counted as a false ID. In a simultaneous lineup, eyewitnesses view an array of (typically, six) faces presented all at once and are tasked with identifying who they believe to be the suspect. In a sequential lineup, faces are presented one at a time and eyewitnesses are asked to either identify the face as the suspect and terminate the lineup or reject the face and view the next face in the sequence. This continues until the suspect is identified or all faces are rejected.

Lindsay and Wells (1985) were the first to compare the sequential and simultaneous lineup procedures. In their study, participants given a simultaneous lineup had a correct ID rate of 0.58 and a false ID rate of 0.43. Participants shown a sequential lineup had a correct ID rate of 0.50 and false ID rate of 0.17. Lindsay and Wells concluded that sequential lineups were superior to simultaneous lineups. To explain this result, Lindsay and Wells suggested that simultaneous lineups promote the use of relative judgments. Wells first proposed this idea, stating that “the term relative judgment refers to the fact that the witness seems to be choosing the lineup member who most resembles the witnesses’ memory *relative* to other lineup members” (Wells 1984, p. 92). According to this idea, witnesses who view all the faces in a simultaneous lineup compare the faces of a lineup relative to each other and choose the member that best matches the witness’s memory.^1^ Relative judgments can be contrasted with an absolute judgment strategy, in which witnesses compare faces (typically in a sequential lineup) directly to memory rather than to each other. Lindsay and Wells concluded that a relative judgment strategy is not necessarily harmful in target present lineups, but leads to higher rates of false IDs in target absent lineups. The authors further argued that a sequential lineup would reduce the propensity to use a relative judgment strategy and that this would result in a lowering of the false ID rate but would have little adverse effect on the correct ID rate. This led to their recommendation to use the sequential lineup in real-world settings in order to protect the innocent from being chosen from lineups. This recommendation has been accepted by many policymakers, as has the relative judgment theory that supports it (Innocence Project 2015; Wells et al. 1998).

The goal of this paper is to elucidate how eyewitness memory is mediated through the exploration of formal conceptualizations of recognition memory and, as a result, be better equipped to evaluate theories such as the relative judgment theory advocated by Wells and colleagues. As we shall see, the language of Lindsay and Wells’s (1985) relative judgment theory, and Wells, Steblay, and Dysart’s (2012) update, appear to invoke two different conceptualizations of recognition memory: discrete-state and continuous mediation. We begin with a short description of each. We then outline the tenets of the relative judgment theory that fit into each of these conceptualizations and propose one possible interpretation that is consistent with the language of the theory. Next, we report two studies that empirically evaluate this interpretation. Based on our evidence, we will then re-evaluate the relative judgment theory and the broader implications of our results for theories of eyewitness ID.

### Discrete-state and continuous models of recognition

One class of models of recognition memory is consistent with discrete-state mediation conceptualizations of recognition memory (e.g. Rouder & Morey 2009). This conceptualization assumes that items can be in one of two states in memory that affect their probability of being recognized and classified as “Old” or “New.” The simplest model of discrete-state mediation is a single high-threshold (1HT) model. Under this model, Old items can be in either a detect or guess state. In the detect state, Old items are able to be correctly classified as “Old.” If an item is not detected as “Old,” it enters a guess state in which no mnemonic information about the item is available, and it can either be guessed as “Old” or guessed as “New.” According to the 1HT model, New items can only enter a guess state, from whence it can be guessed “Old” (a false alarm) or guessed “New” (a correct rejection).

**Fig. 1:**
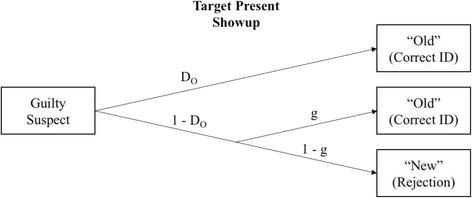
Show-up decision process under the 1HT model. In a target present show-up, the guilty suspect can be detected as “Old,” a correct ID, or guessed as “Old,” a correct ID, or “New,” a false rejection

To extend the conceptualization of discrete-state mediation to the eyewitness memory situation, consider the show-up situation, where only one face is presented to the witness. A witness is tasked with either identifying the face as the suspect or rejecting the face. The Old item in this case would be a guilty suspect. When presented a target present show-up (which includes the guilty suspect), the witness will either detect the guilty suspect as “Old” (and make a correct ID with probability D_O_) or fail to detect the suspect and guess, either guessing that the suspect is “Old” and making a correct ID (with probability *g*), or guessing that the suspect is “New” and rejecting the lineup (with probability 1 – *g*) (see Fig. 1). No mnemonic information is available to guide guessing, only a response bias to endorse “Old” versus “New.” Only guessing governs responses during a target absent show-up.

**Fig. 2:**
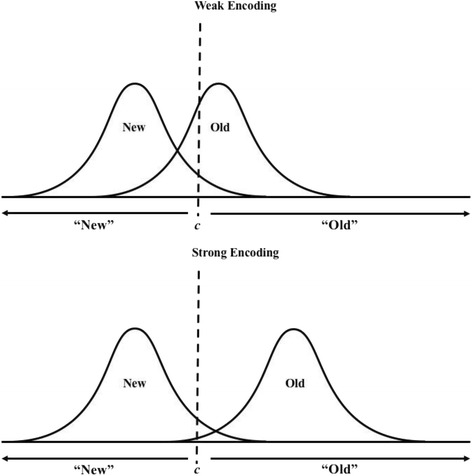
Depiction of SDT. New and Old items fall in two separate distributions of strength values. Tested items that fall above the criterion value (*c*) will be labeled “Old” and those that fall below *c* will be labeled “New.” The *top panel* indicates a situation in which Old items have been encoded weakly, indicated by the large degree of overlap of the New and Old distributions. The *bottom panel* indicates a situation in which Old items have been encoded strongly and the distributions have separated further

The second model of interest invokes continuously mediated processes underlying recognition memory. We focus on signal detection theory (SDT; Green & Swets 1966; Macmillan & Creelman 2005) as an exemplar of this class. SDT assumes that all items, both Old and New, possess latent strength values. These strength values vary continuously and are commonly depicted by normal distributions, whereby most items have similar (average) levels of strength, but some items are very strong and some are very weak. When items are studied, the strengths of these items increase, and the distribution of Old item strengths shifts, resulting in two overlapping distributions of strengths that characterize New and Old items. These distributions can vary in degree of overlap depending on how well Old items have been encoded (see Fig. 2). At study, the strengths of tested items are compared to a decision criterion (*c*). Items that fall above *c* are classified as “Old” and items that fall below *c* are classified as “New.”

To illustrate, consider again the show-up. When an eyewitness is presented with a guilty or innocent suspect, SDT posits that a suspect that falls above *c* will be classified as “Old” and a suspect that falls below *c* will be classified as “New.” If a guilty suspect in a target present show-up falls above *c*, it will be classified as “Old” and the witness will make a correct ID. If the guilty suspect falls below *c*, it will be classified as “New” and the witness will make a false rejection. Conversely, if an innocent suspect from a target absent show-up falls above *c*, it will be classified as “Old” and the witness will make a false ID; if it falls below *c*, it will be classified as “New” and the witness will make a correct rejection.

### Mediation of relative judgment theory

Because the relative judgment theory is a verbal rather than formal theory, there are no explicit references to the recognition models we just outlined. In fact, past descriptions of the theory imply both discrete and continuous mediation. For example, Wells et al. (2012) argued “the higher rate of hits from the simultaneous lineup is actually just the result of *lucky guesses* stemming from a higher rate of choosing” (p. 268, emphasis added). For a similar idea, see Penrod, Garcia, and Robertson (2005). This language suggests discrete-state mediation because no mnemonic information is assumed to be attached to these non-detected stimuli. In support of this discrete-state interpretation, Clark (2012) wrote, regarding the relative judgment strategy, that “…it assumes an all or nothing theory of memory, in which the witness makes a recognition decision based on a true memory, or he or she simply guesses…” (p. 281). Moreover, according to Wells et al., relative judgments occur when “…the witness is unable to answer the difficult question (‘Is this the culprit’) and instead shifts to an easier question (‘Which is the closest?’)” (p. 268). One could conceptualize the “shift” from asking who the culprit is to who is closest, as similar to failing to detect the target in a basic recognition task. But Wells (1984) stated that “absolute processing implies that a match (i.e., between a lineup member and one’s recollection of the perpetrator) must exceed some cut-off in order to produce an identification response” (p. 95). This language is reminiscent of a continuously mediated model because absolute judgments appear to involve the comparison of lineup members to memory, much as a strength value is compared to a criterion in SDT. In fact, using SDT, Wixted and Mickes (2014) argued that the relative judgment theory reflected changes in response bias, or the willingness to choose a suspect from a lineup. Specifically, when faces are presented in isolation (sequentially), there is less pressure to choose the face and a more conservative criterion is adopted. However, when faces are presented simultaneously, there is more pressure to choose and the criterion is pushed to a more liberal level, increasing the likelihood that a filler is chosen. Response bias refers to placement of a criterion, and is a basic tenet of SDT; this idea does not fit a discrete-state conceptualization because there is nothing to weigh evidence against in discrete models.

Because different iterations of the relative judgment theory contain facets of discrete-state and continuous mediation, we decided to consider one possible formal implementation of the theory, which would then allow us to develop a hypothesis to test. We chose a dual-process framework, specifically the model proposed by Yonelinas (1994), and present the relative judgment theory in that context. According to the dual-process model, recognition decisions can rely on familiarity or recollection. Recollection is an all-or-none process in which a specific memory of studying an item either succeeds or fails at being retrieved.^2^ The recollection process in the dual-process model is discrete, analogous to the detection process of the 1HT model. We will not test this assumption because our focus is on non-recollected decisions and how they are mediated. There are two possibilities regarding how these decisions are mediated.

One possibility, consistent with Yonelinas (1994), is that items that are not recollected can be recognized based on a continuous, familiarity process that operates under the assumptions of SDT. This seems in line with the weighing of memorial evidence of the lineup members against each other (Lindsay & Wells 1985), which would be a diagnostic process.^3^ Alternatively, if the eyewitness relies on a guessing process (Penrod et al. 2005; Wells et al. 2012), it implies that the relative/familiarity judgment is discrete, like the 1HT model, which means that the guessing process relies on zero mnemonic information (a “lucky guess”). Therefore, relying on a relative judgment strategy in this situation provides no diagnostic information to the witness. The lineup member that the witness believes looks most like the perpetrator is no more likely to be the actual perpetrator than any of the other lineup members. Therefore, the decision process would be completely random, as if rolling a die, and choosing the actual perpetrator would be a lucky guess. In this case, even though the decision process is subjectively continuous to the witness (i.e. he or she is choosing the person that is “most like” the perpetrator), it can nevertheless be modeled by a non-diagnostic, discrete-state process like the 1HT.

To reiterate, our dual-process interpretation is not the only way to instantiate relative judgment theory, but it does allow for a test regarding how the information involving non-recollected stimuli is mediated. According to the dual-process view of relative judgment theory, non-recollected faces in a simultaneous lineup would be subject to a relative judgment process. This process could be continuous, and therefore diagnostic of guilt, or it could be discrete, implying a non-diagnostic guessing process. We will empirically test discrete-state and continuous model predictions using a task similar to eyewitness ID. In doing so, we aim to better understand the recognition evidence that mediates these decisions so as to better inform theory.

### Empirical evidence for discrete-state and continuous mediation

Discrete-state and continuous models make different predictions about the shape of receiver operating characteristic (ROC) curves. ROC curves are constructed by plotting the hit rate and false alarm rate at each level of response bias (i.e. willingness to label an item as “Old”). Response bias is often assessed using confidence judgments (Wixted 2007; Yonelinas & Parks 2007), with high confidence signaling a more conservative response bias and low confidence signaling a more liberal response bias. Discrete-state mediation predicts linear ROCs, whereas continuous mediation predicts curvilinear ROCs. However, empirical evidence from the basic recognition memory literature almost always produces curvilinear ROC curves (Wixted 2007), supporting a continuous mediation of recognition memory.

Our dual-process view of relative judgment theory, likewise, makes predictions regarding the shape of eyewitness ROC curves. If non-recollected stimuli possess zero mnemonic information (discrete), the ROC will be linear. However, if the non-recollected stimuli are continuously mediated, a curvilinear ROC will result. Recent studies reporting ROCs of simultaneous lineups appear to show curvilinear ROCs (e.g. Gronlund et al. 2012; Mickes 2015; Wetmore et al., 2015). However, there are two problems with relying on ROC shape to differentiate continuous and discrete mediation accounts.

One problem is that eyewitness ROC curves are truncated because they involve plotting only suspect IDs (see Gronlund, Wixted & Mickes 2014; **Wixted & Mickes, 2012). This truncation can make it challenging to assess the shape of the ROC curve. The second problem is that the predictions made by discrete-state models can mimic curvilinear ROC curves (e.g. Broder & Shutz, 2009; Malmberg, 2002; Province & Rouder 2012; but see Chen, Starns, & Rotello 2015). Province and Rouder refer specifically to the effect of relaxing the certainty assumption (Luce 1963). The certainty assumption posits that under discrete-state mediation, all “detect” items are recognized with high confidence and only “guess” items can be recognized with a range of low to high confidence. This assumption leads to the linear ROCs that the model predicts. However, Province and Rouder showed that if you relax the certainty assumption and allow for the possibility that detected items can be recognized with a broader range of confidence, a discrete-state model can indeed predict curvilinear ROCs. Recently, Kellen, Erdfelder, Malmberg, Dube, and Criss (2016) showed that an alternative discrete model (the low-threshold model, Luce 1963), which assumes that New items can exceed a threshold for detection, also can approximate empirical ROC curves. Therefore, ROC analysis is unable to definitively test between discrete-state and continuous mediation.

If ROC analysis will not distinguish between continuous and discrete mediation, another measure is needed. Kellen and Klauer (2014) provided one such measure. In their study, participants were presented with a list of 270 words; 135 words were presented once (weak encoding, W) and 135 words were presented three times (strong encoding, S). At test, participants were presented with three-word, target present arrays (their Experiment 2) and were told to rank each of the words from “Most likely to have been seen before” to “Least likely to have been seen before.” The critical measure was the conditional probability that the actually-studied target of the array was ranked second, given that it was not chosen as most likely to have been seen before (c_2_).

The c_2_ measure requires minimal assumptions (in contrast to ROC analysis). For example, the certainty assumption has no effect on c_2_. Moreover, c_2_ evaluates the most fundamental prediction of discrete and continuous models. In a discrete-state model, although strong items would be more likely to be ranked first (D_O_^S^ > D_O_^W^), if a strong target was not identified as old, it would have an equal likelihood of being ranked second or third because judgments regarding these items must arise from the guess state. In a guess state, the amount of mnemonic information is zero, regardless of whether the tested item actually was strong or weak. This leads to equal c_2_ predictions for strong versus weak items, even though the average hit rate of strong items (strong items ranked first) would be greater than the average hit rate of weak items. According to a continuous model, however, strong items, on average, have a greater strength than weak items. Therefore, if a strong target was not ranked first, it would nevertheless have a greater likelihood of being ranked second than a weak target because it would fall higher (on average) in the target distribution (see Kellen & Klauer 2014, for proofs). In sum, the predictions regarding c_2_ under a discrete-state model are: c_2_^S^ = c_2_^W^, but predictions regarding c_2_ under a continuous model are: c_2_^S^ > c_2_^W^. Using words as their stimuli, Kellen and Klauer (2014) found evidence supporting a continuous model (c_2_^S^ = 0.63 > c_2_^W^ = 0.55, Exp. 2).

In order to evaluate our interpretation of the relative judgment theory, we utilized the same paradigm and c_2_ measure as Kellen and Klauer (2014) to test whether memory for faces is mediated by continuous or discrete processes. There is theoretical and practical merit in using faces rather than words as our critical stimuli. For example, words are processed and encoded differently than faces. Olivares, Iglesias, and Rodriquez-Holguin (2003) found evidence of separate linguistic and non-linguistic event-related potentials when comparing the N400 component using facial and non-facial stimuli, which suggests that the brain processes these stimuli differently at the neural level. Additionally, separate, specialized modules used in the processing of faces and visual words have been identified using fMRI (Kanwisher 2010). These studies suggest that recognition and processing of faces and words are not homologous at all levels. Additionally, eyewitness tasks, inside and out of the laboratory, require encoding and recognizing novel faces, which differs from encoding and recognizing known words.

Kellen and Klauer (2014) found evidence of continuous mediation for words in a ranking task, adding to the body of ROC evidence suggesting that recognition memory is driven by continuous mediation. Experiment 1 could replicate these findings and extend Kellen and Klauer’s results to faces. Alternatively, the evidence indicating that faces are processed differently from words suggests the possibility that recognition of faces may be mediated differently than words. According to the dual-process view of the relative judgment theory, evidence of continuous mediation would signal that relative judgments (in the absence of recollection) involve the weighing of diagnostic memorial evidence (like SDT), but evidence of discrete-state mediation would be indicative of a reliance on a non-diagnostic guessing process (as in the 1HT). Experiment 1 sought to elucidate the use of discrete-state or continuous mediation using faces as the critical stimuli.

#### Experiment 1

## Method

### Participants

Participants were 53 undergraduates who participated in the study in exchange for course credit in an introductory psychology course.

### Procedure

Faces were arbitrarily chosen to be either targets or fillers and all 53 participants saw the same targets at study. During the study phase, participants were instructed that they would be presented with a series of faces, one after another, and should try to memorize each face. After indicating they understood the instructions, participants viewed 100 male Caucasian faces (aged 20–40 years) for 1000 ms each, separated by 500 ms inter-stimulus fixation crosses. Fifty of the faces were presented once and 50 were presented a total of three non-sequential times, for a total of 200 study events. All 200 events were presented in a random order and conditions were counterbalanced between even and odd numbered participants such that odd numbered participants studied faces once that even numbered participants studied three times and vice versa.

Following the study phase, participants completed 20 arithmetic problems as a distractor. Participants were instructed that three two-digit numbers would be presented to them and that the first two numbers may or may not add up to the third. Participants were told to press “Y” on their keyboard if the first two numbers added up to the third number, or “N” if it did not. After indicating they had read and understood the instructions, participants viewed the 20 distractor problems.

Following the distractor, participants began the test phase. Participants were instructed that they would be presented with an array of three faces, only one of which had been studied before (the target), and that the position of the studied face would be random. Instructions indicated that the participants were to rank each face from 1st (most likely to have been seen before) to 3rd (least likely to have been seen before). Once participants indicated that they had read and understood the directions for the test phase, the arrays were presented.

Each array consisted of three faces presented in a row in the middle of the screen with three check boxes under each face that read “1st,” “2nd,” and “3rd.” Instructions at the top of the screen reminded participants to rank each face from 1st (most likely) to 3rd (least likely) and to only provide one rank per face. After checking the appropriate ranking under each face, participants were allowed to change their rankings until they indicated that their rankings were final and moved on to the next array. Each participant completed a total of 100 test trials, 50 with weak targets and 50 with strong targets.

## Results

In order to verify that the encoding manipulation worked, we compared the average hit rate (i.e. targets ranked 1st) for the weak targets to the average hit rate for the strong targets using a dependent *t*-test. The average hit rate of the strong targets (*M* = 0.71, *SD* = 0.16) was significantly greater than the average hit rate of the weak targets (*M* = 0.52, *SD* = 0.15) (*t*(52) = –10.38, *p* < 0.001). This indicates that faces studied three times were encoded better than those studied once. Proportions of the target ranked 1st, 2nd, and 3rd were calculated for each participant and the means are reported in Table 1.

**Table 1:** Proportion of weak and strong targets ranked 1st, 2nd, and 3rd and c_2_ values for Experiments 1 and 2

Exp. 1	1st	2nd	3rd	c_2_
Weak	0.52	0.26	0.22	0.55
Strong	0.71	0.18	0.12	0.62
Exp. 2	1st	2nd	3rd	c_2_
Weak	0.45	0.31	0.23	0.57
Strong	0.61	0.24	0.15	0.61

Critically, we were interested in the conditional probability of targets ranked 2nd given they were not ranked 1st. Consequently, c_2_ was calculated for strong and weak conditions for each participant. A dependent *t*-test indicated that average c_2_^S^ (*M* = 0.62, *SD* = 0.15) was significantly greater than average c_2_^W^ (*M* = 0.55, *SD* = 0.11) (*t*(52) = 2.82, *p* < 0.01). A non-parametric test (Wilcoxon signed-rank test) reached a similar conclusion, indicating that c_2_^S^ was significantly greater than c_2_^W^ (V = 375.5, *p* < 0.01). Cohen’s effect size (*d* = 0.54) indicated a moderate effect. Results were also analyzed using the BEST (Bayesian Estimation Supersedes the *t* Test) analysis developed by Kruschke (2013). BEST uses Bayesian inference to fit data with the *t*-distribution and provides a 95 % highest density interval (HDI) of possible parameter values (i.e. where most of the credible parameter values fall). The 95 % HDI for the mean of c_2_^W^ was (0.52–0.58, mean estimate of 0.552). The 95 % HDI for the mean of c_2_^S^ was (0.58–0.67, mean estimate of 0.623). The 95 % HDI for the mean difference was (0.020–0.12, mean estimate of 0.071). The HDI interval does not include 0, indicating evidence for a non-zero difference between c_2_^W^ and c_2_^S^. Results supported the hypothesis that face recognition is continuously mediated, substantiated by the finding that c_2_^S^ was greater than c_2_^W^.^4^

## Discussion

The findings provide supporting evidence for the continuous mediation of recognition memory for facial stimuli. However, stronger evidence for continuous mediation in eyewitness situations would take the form of continuous c_2_ patterns (c_2_^S^ > c_2_^W^) when there is a mismatch of faces between study and test. When an eyewitness views a crime, the face he or she encodes is not a direct match to the face seen in a subsequent ID procedure. Therefore, it is important to establish that the evidence for continuous mediation in Experiment 1 replicates in a more externally valid situation. We sought to generalize the Experiment 1 results by implementing a mismatch similar to what real eyewitnesses to a crime would experience. To accomplish this, we ran a second experiment in which the same individuals were seen at study and test, but the photos of these individuals differed in facial expression and in the clothing they wore.

### Experiment 2

## Method

### Participants

Participants were 115 undergraduate students (84 women) aged 18–25 years (*M* = 18.6, *SD* = 1.20) who received credit in an introductory psychology course in exchange for participation.

### Procedure

Before the experiment began, participants were presented with a short practice session. We implemented this practice session to ensure that participants understood the task before our critical data were collected. The practice phase exactly matched the study and test phases in all respects except for the use of female target faces and the exclusion of a distractor task. Participants studied five female Caucasian faces (aged 20–40 years) for 1250 ms, with 500 ms inter-stimulus fixation point, followed by two practice test trials with instructions identical to the actual test phase.

Following the practice phase, participants began the experiment. Participants were instructed that they would view a series of faces and should memorize these faces. Once they indicated that they understood the instructions, 100 male faces were presented in a randomized order for 1250 ms each, interspersed with a 500 ms inter-stimulus fixation point. Fifty faces were presented once in the weak encoding condition, and 50 faces were presented three non-sequential times in the strong encoding condition, for a total of 200 study events. There were 30 Caucasian faces and 20 African American faces at each level of encoding. All 100 unique study faces wore smiles and street clothes. The faces rotated through the two encoding conditions as in Experiment 1 and, like Experiment 1, faces were arbitrarily chosen to be either targets or fillers before the experiment was conducted.

After viewing all 200 study events, participants completed a distractor task. The distractor task was identical to Experiment 1 except 40 math questions were completed. Following the distractor and before the test phase began, participants were given the same instructions as in Experiment 1. After indicating that they had read and understood the instructions, participants began the test phase.

**Fig. 3:**
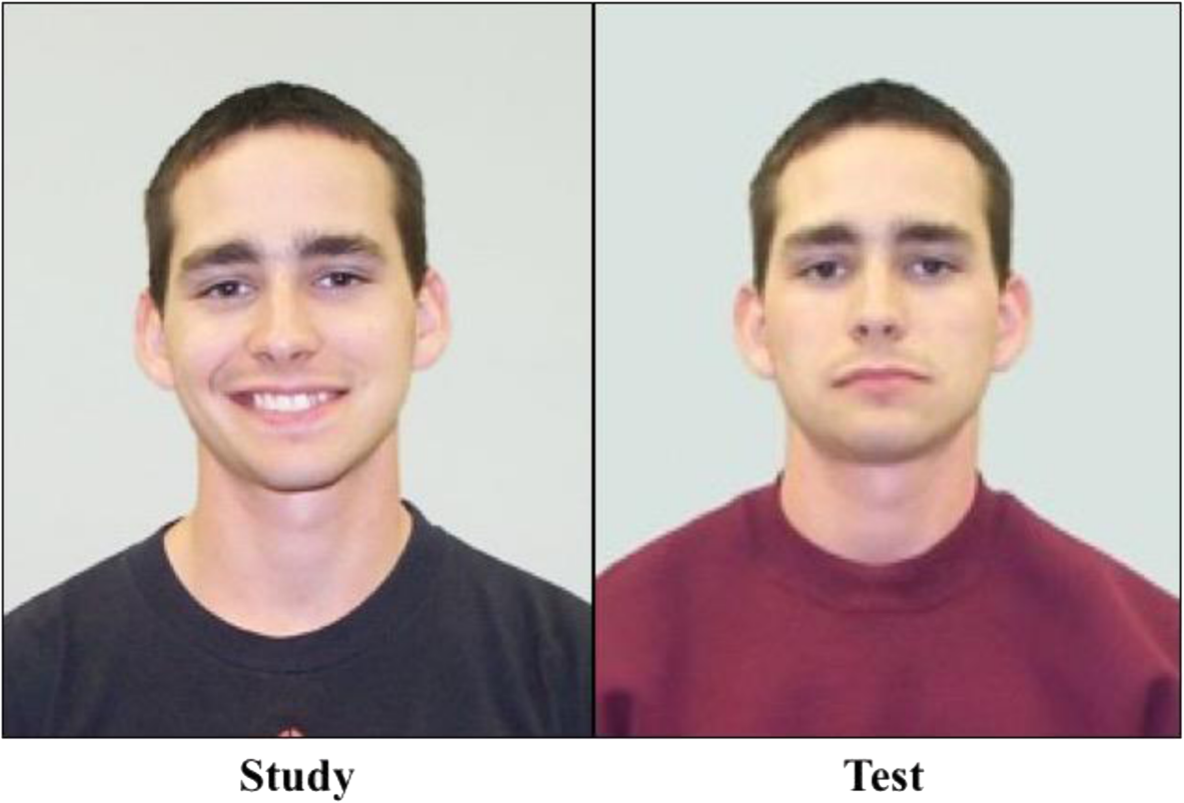
Example of faces used at study and test in Experiment 2. Faces at study (*left*, in figure) were smiling and wearing street clothes. Faces at test (*right*, in figure) had neutral expressions and wore a plain red shirt. Stimuli taken from Meissner, Brigham, & Butz (2005)

Arrays contained one target (either weak or strong) and two new faces presented in a row in the center of the screen. The faces for each array were randomly selected (the target from the pre-determined pool of targets and the fillers from the pre-determined pool of fillers) and the position of the target was randomly determined for each test trial. In addition, Caucasian and African American arrays were presented in a random order. Race never varied within an array. Each face was labeled underneath as “1,” “2,” and “3” from left to right. The faces at test all had neutral expressions (as opposed to happy expressions at study) and wore matching red shirts (as opposed to street clothes at study). See Fig. 3 for a comparison of faces at study and test. Once a face was selected, the number under that face was replaced by “Most Likely,” and participants indicated the face they believed to be next most likely to have been seen before. They were given the option to reset their choices by pressing “0.” Once the second face was selected, it was relabeled “Next Likely,” and the remaining face was relabeled “Least Likely.” Once the rankings were complete, the participants pressed “Y” to continue to the next trial, or pressed “N” and the arrays were reset. After all 100 arrays were ranked, participants were debriefed and dismissed.

### Results

To verify the effectiveness of the encoding manipulation, hit rates of weak and strong targets were compared using a dependent *t*-test. The hit rate for strong targets (M = 0.61, SD = 0.11) was significantly greater than the hit rate of weak targets (M = 0.45, SD = 0.07) (*t*(114) = 15.69, *p* < 0.001). There were no significant differences in hit rates between weak Caucasian faces (*M* = 0.45, *SD* = 0.09) and weak African American faces (*M* = 0.43, *SD* = 0.14) (*t*(114) = 1.67, *p* = 0.10), nor were there significant differences in hit rates between strong Caucasian faces (*M* = 0.61, *SD* = 0.13) and strong African American faces (*M* = 0.60, *SD* = 0.15) (*t*(114) = 0.47, *p* = 0.64). The proportions of targets ranked most, next most, and least likely to have been seen before, are reported in Table 1.

As in Experiment 1, c_2_ measures for weak and strong arrays were calculated for each participant. A dependent *t*-test revealed c_2_^S^ (*M* = 0.61, *SD* = 0.11) was significantly greater than c_2_^W^ (*M* = 0.57, *SD* = 0.09) (*t*(114) = 2.38, *p* = 0.01). A Wilcoxon signed-rank test revealed identical results, with c_2_^S^ significantly greater than c_2_^W^ (V = 2379, *p* = 0.02). Cohen’s effect size (*d* = 0.31) indicated a small to medium effect. Using the BEST analysis, the 95 % HDI for the mean of c_2_^W^ was (0.556–0.590, mean estimate of 0.573). The 95 % HDI for the mean of c_2_^S^ was (0.586–0.627, mean estimate of 0.606). The 95 % HDI for the mean differences was (0.007–0.061, mean estimate of 0.033), which does not include 0, indicating evidence for a non-zero difference between c_2_^W^ and c_2_^S^_._

## Discussion

Experiment 2 replicated Experiment 1 (albeit with a smaller effect), further supporting a continuous model of memory in faxcial recognition. By using a mismatch of faces between study and test to better approximate an eyewitness decision, this experiment provides a more robust test of the mediation involved in facial recognition, which allows for a better evaluation of the relative judgment theory. That is, evidence for continuous mediation using a mismatch paradigm suggests that eyewitness identification tasks engage continuously mediated processes. However, it is interesting to note that the modification that better approximates the eyewitness situation (i.e. mismatch of faces at test and study in Experiment 2) mitigated the effect size and Bayesian evidence. This finding warrants further investigation and additional research using closer approximations to the true eyewitness task, an issue we will explore in more detail in the General discussion.

### General discussion

The goal of these experiments was to better understand the relative judgment theory proposed by Lindsay and Wells (1985) and Wells et al. (2012) in light of evidence for discrete-state or continuous mediation in eyewitness-like paradigms. The theory is important to examine because of its proliferation in the eyewitness literature and its impact on policy decisions. However, because the theory is verbally specified, it is challenging to tie formal models of recognition memory to it, as evidenced by the fact that both continuous and discrete-state mediation appear germane to different instantiations of the theory. We chose to reconcile this problem using a dual-process interpretation, where upon the failure of recollection (and the reliance on relative judgments), eyewitnesses may either guess among alternatives (a discrete, non-diagnostic process), or compare continuous evidence among alternatives (a diagnostic process). The current studies found evidence of continuous mediation in an eyewitness-like situation using the simultaneous presentation of options. Our evidence suggests that continuous evidence meaningfully mediates simultaneous lineup decisions and that according to a dual-process implementation, a version of relative judgment theory that relies on a discrete recollective process should be coupled with a continuous diagnostic relative-judgment process.^5^

Our findings stand contrary to a reliance on lucky guesses in the absence of recollection (guessing with zero mnemonic information). Consequently, it is discrediting to eyewitnesses, and misleading to policymakers, to state that some ostensibly correct eyewitness decisions arise from a non-diagnostic process. In contrast, according to the continuous mediation perspective, ID decisions are made at differing levels of confidence. Consequently, rather than choosing an ID procedure that purportedly reduces relative judgments (the sequential lineup), policymakers instead should determine the confidence level below which an ID decision is considered too unreliable: not because low-confidence decisions are more likely to have arisen from a guess, but because as confidence decreases, accuracy decreases (e.g. Palmer, Brewer, Weber, & Nagesh 2013; Wixted et al. 2015).

### Other tests of relative judgment theory

Clark’s (2003) WITNESS model has been used to explore other aspects of relative judgment theory. For example, Clark and Gronlund (2015) offered an alternative explanation for the primary evidence offered in favor of a reliance on relative judgments (Wells 1993; see also Clark & Davey 2005). Wells (1993) randomly assigned participants to view either a target present lineup or a target removed lineup (a lineup from which the guilty suspect was removed and not replaced). Wells argued that if witnesses are making absolute judgments, those who could have identified the guilty suspect, had he been present, should reject the target removed lineup because the guilty suspect is not present. However, if witnesses are making relative judgments, those witnesses who could have selected the guilty suspect will exhibit what is referred to as a target-to-foil shift, and instead select the next best option from the target removed lineup. Wells found that most participants did not reject the target removed lineup and instead shifted their choices to the fillers. They interpreted this finding as evidence that simultaneous lineup decisions were made using relative judgments, as defined by relative judgment theory (Lindsay & Wells 1985; Wells 1984). But Clark and Gronlund (2015) used the WITNESS model to fit the Wells (1993) data using an absolute decision process, which suggests that a target-to-foil shift is not proof of the use of a relative judgment strategy in simultaneous lineups. Clark and Gronlund offered an alternative explanation based on continuous mediation. Whenever at least two lineup members have strength values above a decision criterion, then the “next-best” lineup member would be chosen if the best match was removed from the lineup.

It is important to note that the concerns our data have raised regarding the diagnosticity of relative judgments refer to one possible formal implementation of the theory proposed by Wells and colleagues (i.e. one that includes a non-diagnostic guessing process). Our data should not be construed as an indictment of the act of comparing faces in a lineup. In fact, comparing faces may have beneficial effects on eyewitness performance. Wixted and Mickes (2014) proposed a SDT-based model that posits that witnesses use distinguishing, diagnostic features of lineup members to inform their choices. In a simultaneous lineup, witnesses compare among fillers, not because they have shifted to a non-diagnostic process, but in order to eliminate shared features that are not diagnostic and focus on those that are. For example, if all lineup members have brown hair, witnesses should not use that feature to inform their decision. The diagnostic-feature detection theory can also explain how witnesses approach sequential lineups. When faces are not presented all at once, witnesses have a difficult time determining which features are diagnostic. As the sequential lineup unfolds, however, witnesses can begin to discern which features are shared by all lineup members viewed so far (and thus are non-diagnostic) and which features are potentially unique to the perpetrator. This is one reason why sequential lineup performance sometimes matches simultaneous lineup performance (e.g. Gronlund et al. 2012) if the suspect (guilty or innocent) is positioned later in the sequential lineup (Carlson et al. 2008; Gronlund, Carlson, Dailey, & Goodsell 2009; for a similar explanation for sequential position effects, see Goodsell, Gronlund, & Carlson 2010). However, a determination of whether (or when) eyewitnesses make comparative judgments among faces in a lineup can only be made when the component processes are formally specified and clear-cut predictions can be derived.

### Future directions

There is a good deal of variability in the c_2_ measures in our sample (as was the case in Kellen & Klauer 2014, see their Figure Four). Some participants displayed patterns that mimicked discrete-state mediation. Although this could be statistical noise, as would be expected when considering a small portion of trials (i.e. misses), it is also possible that some participants adopt strategies during the ranking procedure that produce discrete-state c_2_ patterns. Recently, Kellen and Klauer (2015) found evidence for discrete-state mediation using a similar paradigm to Kellen and Klauer (2014), except that confidence ratings of individual items replaced the ranking judgments for arrays of items. It appears that the task (confidence ratings rather than ranking judgments) can cause participants to adopt a discrete strategy. A study using confidence ratings should be done to determine if the same pattern holds for faces. This study also would be important given the call for the use of confidence judgments to construct ROC curves to assess eyewitness performance (Gronlund et al. 2015; Wixted & Mickes 2012). Kellen et al. (2016) also found that Luce’s (1963) discrete low-threshold model fit the Kellen and Klauer (2014) ranking data as well as did a continuous SDT model. Clearly, more work needs to be done to understand the c_2_ ranking paradigm, as well as determine what factors, or strategies, affect when recognition memory is found to be continuously or discretely mediated. These include factors that distinguish an eyewitness situation from the typical laboratory study.

Experiment 2 was our first foray into better approximating an eyewitness situation and the mismatch of faces from study to test mitigated the c_2_ differences between strong and weak items. That is, Experiment 1 had a Cohen’s *d* of 0.54 and a 95 % HDI of mean differences (0.020–0.12) that contained a lower estimate further from zero than Experiment 2, which had a smaller Cohen’s *d* of 0.31, a 95 % HDI of mean differences (0.007–0.061), and a lower bound closer to zero. One possible explanation for the decreasing effect size may have arisen due to perceived task differences: Experiment 1 is a simple matching task (the photos from study to test were identical), whereas Experiment 2 requires a participant to make a determination whether this different photo corresponds to the same person studied previously. The matching task in Experiment 1 seems amenable to weighing memorial evidence against a simple criterion (continuous processing). Experiment 2, on the other hand, because it is more difficult cognitively, may (sometimes) engender more than just a matching process. If there is more than simple evidence-weighing going on (e.g. participants’ decisions are confirmed by recollection), it could mitigate the c_2_ results in Experiment 2. More work is needed to understand this possible task complexity effect, which can be achieved, in part, by follow-up experiments that more closely approximate the eyewitness situation.

How will other factors that characterize the eyewitness task influence the degree of continuous versus discrete mediation? For example, the current studies randomly selected the fillers in each array so that each participant would not see the same array composition as other participants. However, in an eyewitness situation, fillers are not chosen at random, but rather matched to the description of the suspect in order to increase lineup fairness. This prevents a suspect from standing out among the fillers. Future studies using the c_2_ paradigm should construct arrays that vary in fairness and evaluate how this factor impacts memory mediation. If the target stands out from the fillers (biased), participants may adopt a discrete-like strategy, whereby the “obvious” target is ranked as first and remaining choices are distributed arbitrarily. In unbiased arrays, on the other hand, where faces closely resemble one another, participants may have to adopt a more continuous strategy in order to differentiate among the faces.

Another way to better approximate the eyewitness situation is to introduce the presence of innocent suspects via target absent arrays. In the current studies, all arrays were target present and participants were aware of this composition. However, the knowledge that some arrays do not contain targets may invite more participants to more consistently adopt continuously mediated strategies, reducing the percentage of participants whose summary data strayed from the average. Fair lineups and target absent situations are commonplace in the eyewitness literature (Clark 2008; Fitzgerald, Price, Oriet, & Charman 2013) and if patterns of c_2_ integrating these variables continue to support continuous mediation of facial recognition, it will further strengthen the case that continuous evidence mediates eyewitness identification.

## Conclusions

The main goal of our studies was to evaluate the relative judgment theory (Lindsay & Wells 1985; Wells 1984; Wells et al. 2012) by mapping the theory onto formal discrete and continuous conceptualizations of facial recognition memory through the use of a dual-process model. We used a critical test developed by Kellen and Klauer (2014) and found evidence for continuous mediation, a finding that requires the re-examination of some of the tenets of the relative judgment theory, in particular, the idea that illegitimate hits are the result of a discrete, or non-diagnostic, guessing process when recollection does not occur. As we have noted, we are not the first to raise concerns about this theory (e.g. Clark & Gronlund 2015). It is our view that a more productive approach toward theorizing in eyewitness ID should involve use of formally specified models (also see Clark 2008) that seek to explain a wide range of eyewitness phenomena (e.g. the WITNESS model, Clark 2003; the diagnostic feature model, Wixted & Mickes 2014). Understanding the mechanisms underlying eyewitness ID is of paramount importance. If we can better understand the processes governing an eyewitness decision, we can better inform policymakers regarding how eyewitness evidence should be collected and utilized by the criminal justice system.

## Footnotes

1 It is important to note that we refer to the term “relative judgment” as the explanation described by Lindsay and Wells, and not the act of comparing faces in a lineup. In fact, as we discuss later, comparing faces can have beneficial effects on eyewitness performance (Wixted & Mickes, 2014). We will refer to the latter as *comparative judgments* to keep this idea separate from relative judgment as an explanation.

2 There are other dual-process models that make different assumptions regarding recollection (e.g. Rotello, Macmillan, & Reeder, 2004; Wixted & Mickes, 2010), but the Yonelinas model fits the language of the relative judgment theory best.

3 Wells et al. (2012) refer to hits made using relative/familiarity processes as *illegitimate* and therefore non-diagnostic, regardless of whether the process is continuous or discrete. This is contrary to what Yonelinas proposed regarding familiarity (unless *d*’ = 0). We believe that a continuous relative/familiarity process would be diagnostic and only a discrete, guessing process should be considered non-diagnostic.

4 All data are available in the Open Science Framework at https://osf.io/rdu2v/.

5 Because the relative-absolute judgment strategy may allow for a mixture of discrete and continuous processes, one could suppose that the c_2_ paradigm will only find evidence supporting discrete mediation when the relevant processes on all trials are purely discrete. In a new experiment similar to Experiment 2, we had participants report their confidence in their first choice. Even when we limited our c_2_ calculation to only those trials in which participants reported being highly confident in the choice ranked first, we found the same c_2_ advantage (c_2_^S^ = 0.63, c_2_^W^ = 0.56). One would think that if discrete mediation was ever occurring, it would occur more often for these high confidence decisions. That is, a participant might reason that, because he or she is certain that the face ranked first is a previously studied face, a participant would arbitrarily assign ranks to the remaining alternatives. But we found this was not the case, suggesting that even arrays involving high-confidence rankings were processed continuously.

## References

[CR1] Broder A, Schutz J (2009). Recognition ROCs are curvilinear - or are they?. On premature arguments against the two-high-threshold model of recognition, Journal of Experimental Psychology: Learning, Memory, and Cognition.

[CR2] Carlson CA, Gronlund SD, Clark SE (2008). Lineup composition, suspect position, and the sequential lineup advantage. Journal of Experimental Psychology, Applied.

[CR3] Chen T, Starns JJ, Rotello CM (2015). A violation of the conditional independence assumption in the two-high-threshold model of recognition memory. Journal of Experimental Psychology. Learning, Memory, and Cognition.

[CR4] Clark, S. E., & Gronlund, S. D. (2015). In J. G. W. Raaijmakers, R. Goldstone, M. Steyvers, A. Criss, & R. M. Nosofsky (Eds.), Cognitive modeling in perception and memory: a festschrift for Richard M. Shiffrin (pp. 245–258). New York City: Psychology Press.

[CR5] Clark SE (2003). A memory and decision model for eyewitness identification. Applied Cognitive Psychology.

[CR6] Clark SE (2008). The importance (necessity) of computational modeling for eyewitness identification research. Applied Cognitive Psychology.

[CR7] Clark SE (2012). Eyewitness identification reform: data, theory, and due process. Perspectives on Psychological Science.

[CR8] Clark SE, Davey SL (2005). The target-to-foils shift in simultaneous and sequential lineups. Law and Human Behavior.

[CR9] Clark, S. E., Moreland, M. B., & Gronlund, S. D. (2014). Evolution of the empirical and theoretical foundations of eyewitness identification reform. *Psychonomics Bulletin and Review, 21*(2), 251–267. doi:10.3758/s13423-013-0516-y 10.3758/s13423-013-0516-y 24258271

[CR10] Fitzgerald, R. J., Price, H. L., Oriet, C., & Charman, S. D. (2013). The effect of suspect-filler similarity on eyewitness identification decisions: a meta-analysis. *Psychology, Public Policy, and Law, 19*(2), 151–164. doi:10.1037/a0030618

[CR11] Goodsell, C. A., Gronlund, S. D., & Carlson, C. A. (2010). Exploring the sequential lineup advantage using WITNESS. *Law and Human Behavior, 34*(6), 445–459. doi:10.1007/s10979-009-9215-7 10.1007/s10979-009-9215-7 20076995

[CR12] Green DM, Swets JA (1966). Signal detection theory and psychophysics.

[CR13] Gronlund SD, Carlson CA, Dailey SB, Goodsell CA (2009). Robustness of the sequential lineup advantage. Journal of Experimental Psychology Applied.

[CR14] Gronlund SD, Carlson CA, Neuschatz JS, Goodsell CA, Wetmore SA, Wooten A (2012). Showups versus lineups: an evaluations using ROC analysis. Journal of Applied Research in Memory and Cognition.

[CR15] Gronlund SD, Mickes L, Wixted JT, Clark SE (2015). Conducting an eyewitness lineup: How the research got it wrong. Psychology of Learning and Motivation.

[CR16] Gronlund SD, Wixted JT, Mickes L (2014). Evaluating eyewitness identification procedures using receiver operating characteristic analysis. Current Directions in Psychological Science.

[CR17] Innocence Project. (2015). Eyewitness Identification. http://www.innocenceproject.org/causes-wrongful-conviction/eyewitness-misidentification. Accessed 1 Dec 2015

[CR18] Kanwisher N (2010). Functional specificity in the human brain: a window into the functional architecture of the mind. Proceedings of the National Academy of Sciences.

[CR19] Kellen, D., Erdfelder, E., Malmberg, K. J., Dube, C., & Criss, A. H. (2016). The ignored alternative: an application of Luce’s low-threshold model to recognition memory. *Journal of Mathematical Psychology*. doi:10.1016/j.jmp.2016.03.001

[CR20] Kellen D, Klauer KC (2014). Discrete state and continuous models of recognition memory: testing core properties under minimal assumptions. Journal of Experimental Psychology, Learning, Memory, and Cognition.

[CR21] Kellen D, Klauer KC (2015). Signal detection and threshold modeling of confidence-rating ROCs: a critical test with minimal assumption. Psychological Review.

[CR22] Kruschke JK (2013). Bayesian estimation supercedes the t test. Journal of Experimental Psychology: General.

[CR23] Lindsay RCL, Wells GL (1985). Improving eyewitness identifications from lineups: simultaneous versus sequential lineup presentation. Journal of Applied Psychology.

[CR24] Luce RD (1963). A threshold theory for simple detection experiments. Psychological Review.

[CR25] Macmillan, N. A., & Creelman, C. D. (2005). Detection Theory: A User’s Guide (2nd Edition). New York: Lawrence Erlbaum Associates

[CR26] Malmberg, K.J. (2002). On the form of ROCs constructed from confidence ratings, Journal of Experimental Psychology: Learning, Memory, and Cognition, 28, 380–387. doi:10/1037//0278-7393.28.2.380 11911394

[CR27] Meissner CA, Brigham JC, Butz DA (2005). Memory for own- and other-race faces: A dual-process approach. Applied Cognitive Psychology.

[CR28] Mickes L (2015). Receiver operating characteristic analysis and confidence-accuracy characteristic analysis in investigations of system variables and estimator variables that affect eyewitness memory. Journal of Applied Research in Memory and Cognition.

[CR29] Olivares E, Iglesias J, Rodriquez-Holguin S (2003). Long-latency ERPs and recognition of facial identity. Journal of Cognitive Neuroscience.

[CR30] Palmer MA, Brewer N, Weber N, Nagesh A (2013). The confidence-accuracy relationship for eyewitness identification decisions: effects of exposure duration, retention interval, and divided attention. Journal of Experimental Psychology, Applied.

[CR31] Penrod S, Garcia L, Robertson R (2005). Assessing the impact of eyewitness guessing and lineup bias on eyewitness performance.

[CR32] Province JM, Rouder JN (2012). Evidence for discrete-state processing in recognition memory. Proceedings of the National Academy of Sciences of the United States of America.

[CR33] Rotello, C. M., Macmillan, N. A., & Reeder, J. A. (2004). Sum-difference theory of remembering and knowing: a two-dimensional signal-detection model. *Psychological Review, 111*(3), 588–616. doi:10.1037/0033-295X.111.3.588 10.1037/0033-295X.111.3.588 15250777

[CR34] Rouder JN, Morey RD (2009). The nature of psychological thresholds. Psychological Review.

[CR35] Steblay NK, Dysart JE, Wells GL (2011). Seventy-two tests of the sequential lineup superiority effect: a meta-analysis and policy discussion. Psychology, Public Policy, and Law.

[CR36] Wells GL (1984). The psychology of lineup identifications. Journal of Applied Social Psychology.

[CR37] Wells GL (1993). What do we know about eyewitness identification?. American Psychologist.

[CR38] Wells GL, Small M, Penrod S, Malpass RS, Fulero SM, Brimacombe CAE (1998). Eyewitness identification procedures: recommendations for lineups and photospreads. Law and Human Behavior.

[CR39] Wells GL, Steblay NK, Dysart JE (2012). Eyewitness identification reforms: are suggestiveness-induced hits and guesses true hits?. Perspectives on Psychological Science.

[CR40] Wetmore SA, Neuschatz JS, Gronlund SD, Wooten A, Goodsell CA, Carlson CA (2015). Effect of retention interval on showup and lineup performance. Journal of Apllied Research in Memory and Cognition.

[CR41] Wixted JT (2007). Dual-process theory and signal-detection theory of recognition memory. Psychological Review.

[CR42] Wixted JT, Mickes L (2010). A continuous dual-process model of remember/know judgments. Psychological Review.

[CR43] Wixted JT, Mickes L (2012). The field of eyewitness memory should abandon probative value and embrace receiver operating characteristic analysis. Perspectives on Psychological Science.

[CR44] Wixted JT, Mickes L (2014). A signal-detection-based diagnostic-feature-detection model of eyewitness identification. Psychological Review.

[CR45] Wixted JT, Mickes L, Clark SE, Grounlund SD, Roediger HL (2015). Initial eyewitness confidence reliably predicts eyewitness identification accuracy. American Psychologist.

[CR46] Yonelinas AP (1994). Receiver-operating characteristics if recognition memory: evidence for a dual-process model. Journal of Experimental Psychology. Learning, Memory, and Cognition.

[CR47] Yonelinas AP, Parks CM (2007). Receiver operating characteristics (ROCs) in recognition memory: a review. Psychological Bulletin.

